# EatSmart, a Web-Based and Mobile Healthy Eating Intervention for Disadvantaged People With Type 2 Diabetes: Protocol for a Pilot Mixed Methods Intervention Study

**DOI:** 10.2196/19488

**Published:** 2020-11-06

**Authors:** Nazgol Karimi, David Crawford, Rachelle Opie, Ralph Maddison, Stella O’Connell, Peter Shane Hamblin, Ashley Huixian Ng, Cheryl Steele, Bodil Rasmussen, Kylie Ball

**Affiliations:** 1 Institute for Physical Activity and Nutrition School of Exercise and Nutrition Sciences Deakin University Melbourne Australia; 2 Diabetes & Endocrinology Centre Sunshine Hospital St Albans Australia; 3 Department of Medicine-Western Precinct University of Melbourne St Albans Australia; 4 Department of Dietetics, Human Nutrition and Sport La Trobe University Melbourne Australia; 5 Diabetes Education Services Sunshine Hospital St Albans Australia; 6 School of Nursing and Midwifery Deakin University Geelong Australia; 7 Centre for Quality and Patient Safety Research Western Health Partnership Sunshine Hospital St Albans Australia; 8 Faculty of Health and Medical Sciences University of Copenhagen Copenhagen Denmark

**Keywords:** type 2 diabetes, healthy eating, diet, low socioeconomic position, self-management, digitally delivered, internet, website, mobile phone, text message

## Abstract

**Background:**

People of low socioeconomic position (SEP) are disproportionately affected by type 2 diabetes (T2D), partly due to unhealthy eating patterns that contribute to inadequate disease self-management and prognosis. Digital technologies have the potential to provide a suitable medium to facilitate diabetes education, support self-management, and address some of the barriers to healthy eating, such as lack of nutritional knowledge or shopping or cooking skills, in this target group.

**Objective:**

This study aims to test the feasibility, appeal, and potential effectiveness of EatSmart, a 12-week, evidence-based, theoretically grounded, fully automated web-based and mobile-delivered healthy eating behavior change program to help disadvantaged people living with T2D to eat healthily on a budget and improve diabetes self-management.

**Methods:**

EatSmart is a mixed methods (quantitative and qualitative) pre-post design pilot study. Sixty socioeconomically disadvantaged people with T2D aged 18 to 75 years will be recruited. Participants will complete self-reported baseline assessments of their basic demographic and clinical data, dietary intake, dietary self-efficacy, and barriers to healthy eating. They will be provided with login access to the EatSmart web program, which includes six progressive skill-based modules covering healthy eating planning; smart food budgeting and shopping; time-saving meal strategies, healthy cooking methods, modifying recipes; and a final reinforcement and summary module. Over the 3-month intervention, participants will also receive 3 text messages weekly, encouraging them to review goals, continue to engage with different components of the EatSmart web program, and eat healthily. Participants will undertake follow-up assessments directly following the intervention 3 months post baseline and again after a 6-month postintervention follow-up period (9 months post baseline). Feasibility will be evaluated using the number of participants recruited and retained and objective indicators of engagement with the website. Program appeal and potential effects on primary and secondary outcomes will be assessed via the same surveys used at baseline, with additional questions asking about experience with and perceptions of the program. In-depth qualitative interviews will also be conducted 6 months post intervention to provide deeper insight into experiences with EatSmart and a more comprehensive description of the program’s appeal.

**Results:**

The EatSmart website has been developed, and all participants have viewed the modules as of May 2020. Results are expected to be submitted for publication in December 2020.

**Conclusions:**

This study will provide data to address the currently limited evidence regarding whether disadvantaged populations with T2D may benefit from digitally delivered behavior change programs that facilitate eating healthily on a budget.

**Trial Registration:**

Australian New Zealand Clinical Trials Registry, ACTRN12619001111167; https://anzctr.org.au/Trial/Registration/TrialReview.aspx?ACTRN=12619001111167

**International Registered Report Identifier (IRRID):**

DERR1-10.2196/19488

## Introduction

### Overview

Type 2 diabetes (T2D), which is defined by relative insulin deficiency resulting from pancreatic β-cell dysfunction and insulin resistance in target organs [1], is considered the epidemic of the 21st century [2]. T2D is a serious challenge for public health, affecting millions of people worldwide [3]. Globally, the number of people with diabetes has increased four-fold in the past three decades, and it was recognized as the ninth major cause of death in recent years [4,5]. According to the International Diabetes Federation, at the end of 2017, about 425 million people had diabetes, and this number is estimated to reach 628.6 million in 2045 [6]. Moreover, in the year 2018, 318 million adults were diagnosed with impaired glucose regulation that puts them at a high risk of developing diabetes in the future [7].

Individuals of low socioeconomic position (SEP), such as those with lower levels of education, working in low-status occupations, or on a low income, as well as ethnic minority groups, are disproportionately affected by T2D [8]. Unhealthy eating patterns and obesity are key risk factors for T2D [4,9,10]. Previous research has shown that compared with those of higher SEP, people of lower SEP commonly have more unfavorable eating patterns, with diets characterized by lower intake of fruits and vegetables and higher intake of less healthy discretionary foods and beverages [11-13]. While structural barriers, such as limited resources, low accessibility to higher quality foods and cost, contribute to these patterns, nutrition knowledge and skill deficits in cooking, lack of interest in cooking, lower prioritization of health during food selection, and low social support for healthy eating are also well-recognized hurdles with which these groups are struggling [14,15].

Existing initiatives to promote healthy eating among people with T2D include multicomponent face-to-face diabetes self-management classes, community health worker programs, and intensive case-management programs [16]. Traditionally, individuals participate in these programs in medical settings or at community health centers to acquire relevant knowledge about diabetes from clinicians, trained interventionists, or credentialed diabetes educators [17]. While such programs can effectively communicate health information to people with diabetes, they are limited in terms of sustainability and scale [18-22]. Time and access requirements preclude the ability of many individuals to attend multiple educational sessions [23-25]. Such intensive programs also incur high costs related to hiring, training, and maintaining educators, which not all practices are resourced to routinely provide [26].

Driven by advances in web-based technologies and requests by people with T2D for easily accessible information and ongoing support, digitally delivered programs potentially offer a convenient and efficient platform to facilitate the delivery and increase the reach of the self-management support needed by disadvantaged people with T2D [27]. Computers, laptops, and mobile phones or smartphones, hosting mobile applications (apps), are commonly used technologies for delivering digital programs [24]. These programs are secure and cost-effective and allow for the delivery of support in a way that is responsive, transactional, and participatory, and accordingly, more persuasive [17,28]. Furthermore, these programs can be easily distributable since they are not limited to a specific location and can be used in clinics, community health centers, home, or on the move at any convenient time [24,27,29]. With the advent of digital technology programs for health, there have been concerns about creating a digital divide, in which people of low SEP have lower access to the internet and mobile devices compared with higher socioeconomic groups [30]. However, more recent studies have demonstrated that the digital divide is gradually diminishing as the internet and mobile technologies have become more accessible to people from different SEPs [31]. Previous studies with low-income populations, including those with T2D and other chronic conditions, showed that a high proportion of participants knew how to use computers or mobile phones and were willing to expand their knowledge of how to use these technologies and find health information via the internet [32-35]. Moreover, it has been suggested that technology can efficiently play a role in bridging the digital divide and serve traditionally “hard to reach” populations. Thus, technology may provide a unique opportunity to decrease health disparities in underserved populations [31].

Previous reviews suggest that digital-based interventions show promise in increasing knowledge and improving diabetes self-management activities, including medication adherence, engagement in physical activity, and healthy eating behaviors in individuals living with T2D [22,27,36-40]. For example, in a review by Boren et al [41], 18 of 21 studies, which used computerized learning technologies to engage and empower people in diabetes self-management, showed positive outcomes in improving 49 out of 112 diabetes outcomes. They stated that while patients’ self management-related behaviors are crucial in diabetes control, computerized learning technology programs can play a major role in improving self-efficacy and diabetes self-care [41].

While digital interventions continue to be developed and researched, their potential for improving diabetes self-management among people of low SEP remains poorly investigated [36], and few have explored the efficacy of these interventions specifically on dietary and healthy eating behaviors [25,42-44]. One example is a study by Arora et al [44], who investigated the effects of educational and motivational text messages on improving healthy eating behaviors, exercising, medication adherence, and foot checks. That intervention led to a daily increase in eating fruits/vegetables by 26.5% and improved diabetes self-efficacy and medication adherence in resource-poor, inner-city patients with diabetes. Another example is the eCare We Care program by Moussa et al [25]. In that study, researchers examined the effect of an evidence-based web program on diabetes knowledge development and skill-building in African American adults with low diabetes literacy [25]. Their findings indicated that the program improved the diabetes and nutritional knowledge of participants [25]. Glasgow et al [45] also investigated the effect of an internet-based diabetes self-management program, with and without additional support, on improving healthy eating, physical activity, and medication taking. Their program improved health behaviors significantly compared to usual care over the 12 months (Cohen *d* for effect size .09-.16) [45].

Although these limited studies show promising results in improving dietary behaviors, they were all heterogeneous in scope. For example, they either concentrated on single nutrition-related health outcomes (eg, weight loss or glycemic control), or combined multiple health behaviors (eg, diet, medication adherence, and physical activity), and utilized different modes of intervention delivery (eg, website, text messages). Therefore, study findings are difficult to integrate and interpret, and there is a need for further research into how disadvantaged populations with diabetes may benefit from digital approaches and whether these programs are feasible, appealing, and can produce long-term and sustainable effects. The EatSmart program was developed to address this gap by designing and evaluating a theoretically grounded, evidence-based web- and phone-delivered healthy eating behavior support program to enable disadvantaged people with T2D to strengthen important skills necessary to eat healthily at low cost.

This project is important as it involves a simple and practical approach tailored to the needs of the many people with T2D who struggle to eat healthily in the context of socioeconomic disadvantage. The program may augment existing clinical management by integrating with people’s daily self-management practices of T2D. It also has the potential to reach larger numbers of people with T2D, regardless of geographic location. Moreover, it is designed to be sustainable without intensive ongoing clinical involvement. EatSmart addresses the critical issues of inequities in diabetes-related self-management and associated morbidity.

### Hypothesis

We hypothesize that delivery of the 12-week EatSmart program is feasible, appealing to participants, and effective in improving healthy eating behaviors and related attitudes. Specifically, we hypothesize that EatSmart will lead to high program satisfaction and engagement and improvements in self-reported intakes of vegetables, fruits, and foods aligned with dietary recommendations. It will also reduce self-reported intakes of discretionary noncore foods, increase self-efficacy, and reduce barriers to selecting and preparing a balanced healthy diet.

### Ethics Approval

Ethics approval has been granted by Deakin University’s Human Research Committee and the Western Health Low-Risk Ethics Panel, approval number 49763; version 4, dated 15 March 2019. All participants will be provided with an information sheet explaining the project aim and will be requested to sign a written informed consent form before participating (Multimedia Appendices 1-3).

## Methods

### Study Design

EatSmart is a mixed methods (quantitative and qualitative) pre-post design pilot study, which is appropriate for evaluating the feasibility, appeal, and potential effectiveness of a novel intervention. Pilot studies are crucial for examining the acceptability of new interventions and improving their delivery to support the future success of more intensive and larger randomized controlled trials [46].

EatSmart focuses on people with T2D who attend diabetes clinics in Melbourne’s western suburbs, which, according to the Australian Bureau of Statistics Socioeconomic Index for Areas index of relative advantaged/disadvantaged, comprises a relatively disadvantaged area.

### Study Population

#### Sample Size Estimation

A sample of N=60 (conservatively assuming up to 33% drop-out) has been selected for this pilot study. As this is a pilot study, no formal power calculation was conducted, and this sample size was determined pragmatically to provide adequate data on potential effects on key outcomes, sample variability, recruitment rates, and retention. This number is also adequate for generating rich qualitative and contextual data on intervention engagement, feasibility, and appeal to inform future larger studies.

#### Participant Recruitment

Sixty people with T2D will be recruited from two outpatient diabetes clinics located at Sunshine Hospital in Melbourne, Australia. Participant recruitment strategies involve (1) distribution of program flyers at diabetes clinics’ waiting rooms and to diabetes nurses and specialist offices in Sunshine Hospital; (2) in-person presentations by diabetes educators or researchers at hospital meetings and diabetes-related events; and (3) face to face recruitment of eligible individuals in diabetes clinic waiting rooms by EatSmart researchers.

For postintervention interviews, patients with T2D who participated in EatSmart and completed the 3-month intervention will be invited by telephone, text message, or email to complete a semistructured interview of up to 40 minutes via telephone or Zoom. Recruitment will be successive until no new themes appear (thematic saturation). Up to ten health care providers from Sunshine Hospital diabetes clinics will also be invited by email to take part in a confidential semistructured telephone or Zoom interview of up to 30 minutes.

#### Eligibility Criteria

##### Inclusion

Adults aged 18 to 75 years with a diagnosis of T2D, regardless of duration. Participants will be required to own and use a mobile phone, be familiar with receiving and reading text messages, and have mobile data allowance to access the internet or a mobile device (phone/tablet) or computer with internet access. Participants will be socioeconomically disadvantaged, as determined by receiving either a Health Care Card or a pension/benefit as the main source of income, or a stated income up to the cut-off levels used to assess eligibility for the Health Care Card. Annual income thresholds for receipt of a Health Care Card in Australia (as of January 2019) are equal to AU $29,172 (US $20,645) for single adults without children, AU $50,388 (US $35,660) for couples, AU $52,156 (US $36,910) for couples with one child and AU $1768 (US $1251) for each extra dependent child. Due to this study’s focus on people from a lower SEP, assessing eligibility can be a sensitive issue. To be respectful when enquiring as to whether or not an individual receives a Health Care Card or low income (ie, is of a low SEP background), a written document with all relevant questions relating to eligibility will be handed to potential participants. This document allows them to check the questions silently in advance, and if they confirm their willingness to answer, the researcher will approach them to complete the recruitment process.

##### Exclusion

Participants will be excluded if they cannot read or speak English; cannot use mobile text messaging or internet; are pregnant or breastfeeding; are visually or hearing-impaired; have an eating disorder; have compounding comorbidities (like clinical depression), as diabetes management is typically more complex in these cases; are planning to have surgery; or are planning to travel for an extended time in the next nine months.

### Intervention

EatSmart is a 12-week evidence-based, theoretically grounded, fully automated healthy eating behavior support program delivered via a website (mobile phone compatible) and text messaging. It is a standalone program that could augment standard diabetes management, which typically involves routine visits and consultation with an endocrinologist and diabetes educator, and sometimes a dietitian (depending on clinical circumstances). If demonstrated to be successful, the EatSmart program could become embedded in clinical practice and promoted by clinical staff as an adjunct treatment option.

The intervention development was informed by Social Cognitive Theory [47], which suggests that people adopt new behaviors through social learning, either through media sources or through imitation of others. Further, it builds on a previous study (“ShopSmart”) by Ball et al [11,48], which trialed a behavior change intervention for increasing purchasing and consumption of vegetables and fruits among women of low SEP and resulted in a significant increase in vegetable consumption during the intervention [11].

EatSmart draws on empirical evidence of the key determinants of eating behaviors in people of low SEP, including previous studies suggesting the importance of addressing perceived cost barriers, food planning, budgeting, preparation skills, and nutrition knowledge [49-52].

Intervention mapping was conducted [53] for the planning and development stage to confirm that this program is based on a strong empirical, theoretical, and practical foundation. A literature review identified existing digital and nondigital approaches to increase vegetable and fruit consumption in people of low SEP that addressed the key constructs of Social Cognitive Theory. The findings of the literature review and intervention mapping, in addition to the experiences gained from the ShopSmart study [11], suggested that focusing on skill building for eating healthy and budgeting wisely can be effective strategies.

The research team has developed a set of modules, all of which focus on the specific needs of people of low SEP, particularly affordability and nutrition-related skills. The materials target the proposed theoretical mediators of nutrition self-efficacy, perceived affordability, and other perceived barriers to vegetable and fruit consumption. The intervention components share similarities with those used in the ShopSmart study [11,48]; however, EatSmart materials are targeted for people with T2D. For example, more focus has been placed on encouraging the consumption of leafy vegetables or fruits with a lower glycemic index.

The skill-based modules of the EatSmart website address the following topics:

1. Importance of vegetables, fruits, and whole foods for health
2. Strategies to buy healthy foods on a budget (highlighting the range of low-cost vegetables and fruits available and their uses, and also the value of more affordable options such as tinned and frozen vegetables and fruits)
3. Planning for smart shopping and how to choose healthy foods, with supermarket shopping tours and advice on label reading
4. Trying and incorporating new vegetables, fruits, and whole foods
5. Building cooking confidence and skills by including recipes of healthy and inexpensive meals from various countries and cultures featuring vegetables and fruits; meal planning; simple and convenient meal and snack ideas; and how to modify recipes to be healthier
6. Storing, preparing, and cooking fresh produce, reducing food waste, and “eating out Smart.”

The website also offers a range of practical activities, including calculating current and ideal spending on different food groups, videos (eg, virtual shopping tours), and links to other resources (eg, government or not-for-profit health information websites).

EatSmart involves delivering key behavior change techniques, including setting goals for purchasing and consuming key food groups, self-monitoring consumption, engaging social support from family and friends, and problem-solving key barriers to healthy eating. A behavior change taxonomy was used to describe the active ingredients of the intervention [54]. The intervention components and their related BCTs are presented in (Table 1).

As part of EatSmart, participants receive three unidirectional text messages/week (36 in total), at a time they indicate is convenient, to encourage them to maintain healthy eating, review their goals, and continue to engage with different components of the website (Multimedia Appendix 4). Multiple behavioral change techniques, including goal setting on core food group intake, informing about health and social (cost) consequences, instructions on how to perform behaviors, and self-monitoring behavior, were employed to design the text messages.

After consenting to the study, participants complete baseline assessments immediately in person or at a more convenient time by telephone or through a secure weblink. The baseline evaluation requests information on basic demographic and clinical data, dietary intake, dietary self-efficacy, and barriers to healthy eating. Upon completing baseline assessments, participants are shown how to log on to the website using their username and password. Once logged in, participants can access the six intervention modules offered to them consecutively on a biweekly basis. Each module can be completed in around 15 minutes, but no recommendation or limitation regarding timing and usage will be given. Therefore, they can read the materials and engage with the website in a suitable and convenient timeframe.

Following intervention completion at three months, participants undertake the same baseline evaluation (except for demographic details). During the 6-month follow-up period (3 to 9 months post baseline), participants will have free access to all website materials; however, they will not receive text messages. After the 3-month data collection period, all participants who complete the postintervention assessments (regardless of their level of engagement) will receive an AU $50 (US $35) shopping voucher as compensation for their time. Those who complete the online follow-up survey will receive an AU $20 (US $14) shopping voucher, and those who agree to be interviewed will receive an extra AU $20 (US $14) shopping voucher.

**Table 1:** Summary of intervention components, targeted determinants, and behavior change techniques.

Intervention component	Primary message/resources (web + text message)	Targeted determinant	Behavior change techniques
Module 1	Importance of a balanced diet for health in diabetes Addressing cost and time barriers to healthy eating Resources for setting healthy eating goals	Knowledge Health values Perceived affordability Perceived barriers: time	Action planning Discrepancy between current behavior and goal Goal setting on core food group intake (behaviors) Information about health consequences Information about social consequences (cost) Instructions on how to perform behaviours Problem-solving Self-incentives Self-monitoring behaviour Self-rewards for goals
Module 2	Planning for healthy eating on a budget	Knowledge and skills (budgeting, planning) Self-efficacy Perceived affordability	Action planning Demonstration of the behavior Discrepancy between current behavior and goal Information about social consequences (cost) Instructions on how to perform behaviors Problem-solving Reviewing behavior goals Self-incentives Self-monitoring behavior Self-rewards Social rewards for goals
Module 3	Planning and shopping smart Label reading Affordability: focus on tinned/frozen options (eg, fruit, vegetables, fish, legumes) Saving money and time Food safety (to reduce waste)	Knowledge and skills Perceived affordability Self-efficacy Perceived barriers: social (family preferences), time	Action planning Conserving mental resources (wallet label reading cards) Demonstration of the behavior Discrepancy between current behavior and goal Goal setting (behaviors) Identification of self as a role model Information about health consequences Information about social consequences (cost) Instructions on how to perform behaviors Practical social support Prompts/cues Self-incentive Self-reward Social reward for goals
Module 4	Confidence in the kitchen Storing and cooking fruit and vegetables Reducing waste Cooking confidence and skills Recipe modification Healthy entertaining	Knowledge and skills Self-efficacy Perceived affordability	Action planning Behavior substitution (recipes, takeaway food) Demonstration of the behavior Discrepancy between current behavior and goal Goal setting (behaviors) Information about social consequences (cost) Instruction on how to perform behaviors Practical social support Problem-solving Reviewing behavior goals Self-incentive Self-reward Social reward for goals
Module 5	Eating out Trying new fruits & vegetables	Skills Self-efficacy Perceived affordability Perceived barriers: taste	Action planning Behavior substitution (eating out) Demonstration of the behavior Discrepancy between current behavior and goal Goal setting (behaviors) Instruction on how to perform behaviors Problem-solving Self-incentive Self-reward Social reward for goals
Module 6	Message reinforcement	Knowledge Skills Self-efficacy Perceived affordability	Conserving mental resources Discrepancy between current behavior and goal Instruction on how to perform behaviors Problem-solving Reviewing behavior goals Self-monitoring behavior

### EatSmart Website Special Design Characteristics

**Figure 1:**
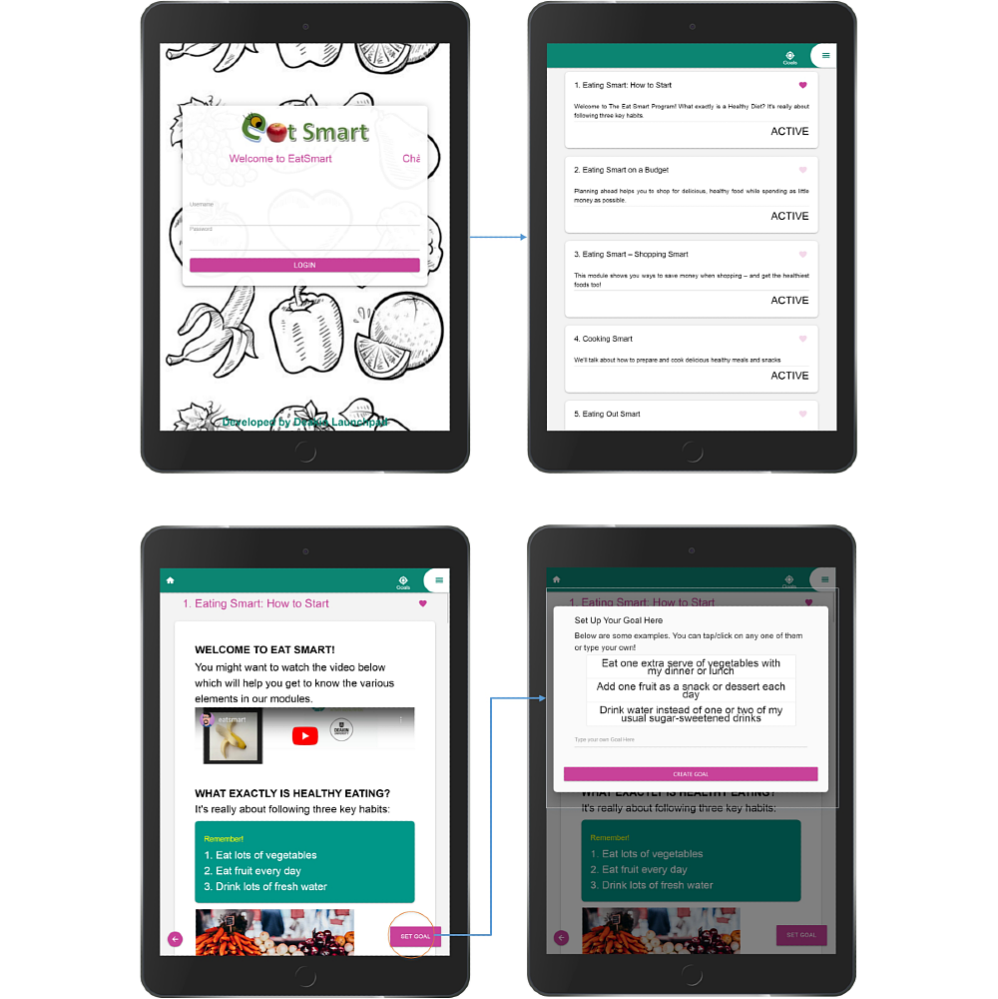
Screenshots of EatSmart website.

**Figure 2:**
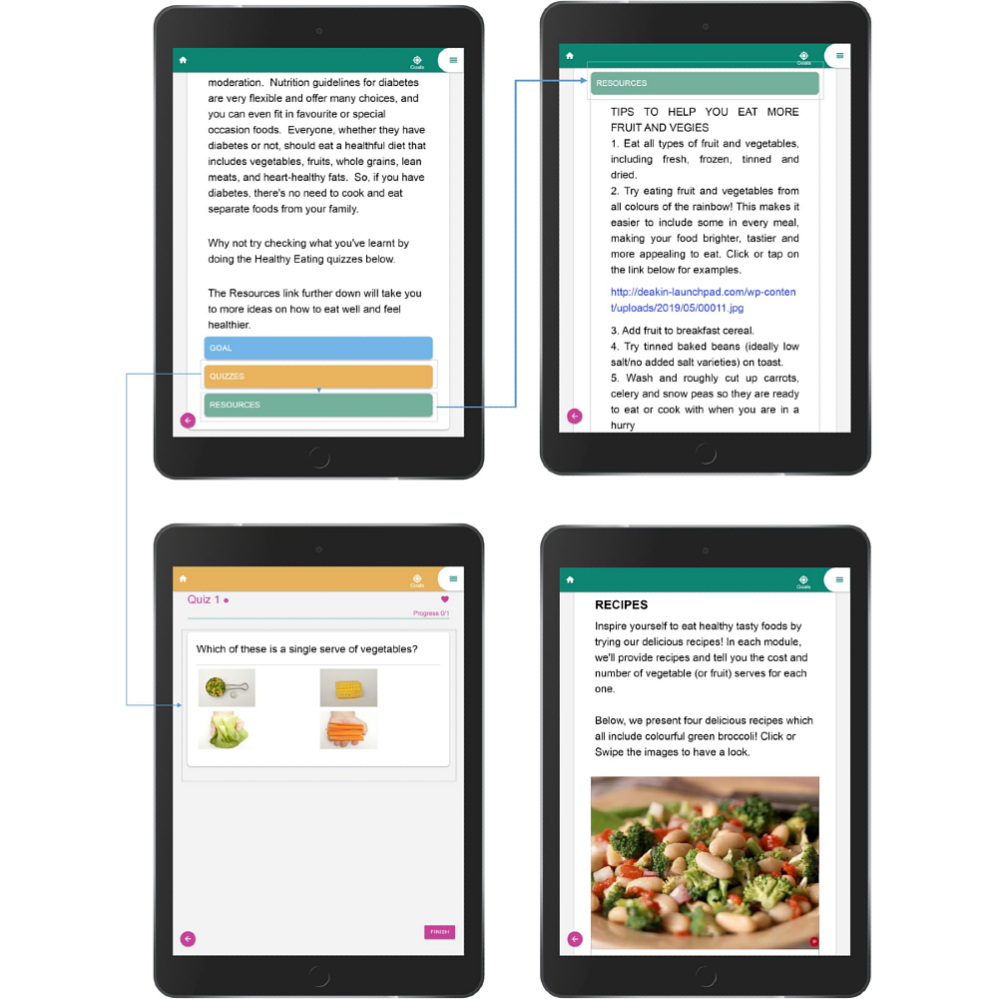
Screenshots of different parts of modules.

The website structure has specific design characteristics to address barriers to electronic literacy (e-literacy) [55] so that it is simple and easy to use and omits unnecessary design elements. Furthermore, the elements are arranged in a way that implies importance (visual hierarchy). For example, the most important points about healthy eating habits and take-home messages are arranged at the top of each module (Figures 1 and 2).

The site’s navigation, overall look, and feel are consistent across all pages by using the same color schemes, typefaces, backgrounds, and tone of the text messages. All six modules comprise four parts. The first part includes healthy eating and nutritional information, followed by goals, quizzes, and resources, which are color-coded to distinguish them from other features. This approach was adopted to enhance the usability of the website [56]. The website is compatible with mobile and desktop devices and different operating systems and browsers. A simple nonverbal users’ guide video [57] is provided at the start of the first module to show participants how to interact with different elements of the website, set their preferred time to receive text messages in their profile, select one of the offered goals, or view videos and recipes.

During website development (before the main project begins), the first four modules were pilot tested with 12 people approached in diabetes clinics at Sunshine Hospital who fell into the project demographic. These participants were asked to complete, with the researchers, a brief survey asking questions about how appealing and understandable they found the website and its modules. No identifying data were collected, although the researchers noted the sex and approximate age of participants in case these factors influenced their views on the modules and/or website design and content. Based on this pilot test, specific suggestions from the participants about content and design of the website (for example, introducing more budget-saving strategies, the inclusion of more simple ethnic recipes, more colorful pictures and visual messages, bigger font size, and easier browsing) have been incorporated to improve interest, readability, tone, and layout in the final development of the intervention for end users.

### Outcomes and Measures

This study’s primary outcome is pre-post intervention (baseline versus 3 months) changes in the consumption of vegetables and fruits.

Secondary outcomes are:

1. Six-month follow-up changes in consumption of vegetables and fruits (baseline versus 9-month),
2. Pre-post intervention and 6-month follow-up changes in other dietary indicators aligned with dietary guidelines (wholemeal/whole grain bread, milk, water, and some discretionary items),
3. Pre-post intervention and 6-month follow-up changes in dietary self-efficacy (including perceived skills and confidence),
4. Pre-post intervention and 6-month follow-up changes in perceived barriers to healthy eating (including budget/perceived unaffordability, family preferences, taste, and skills),
5. Feasibility and engagement,
6. Appeal, satisfaction, usability, and longer term acceptability of the program.

Study measures across time points are presented in Multimedia Appendix 5. Demographic data including age, sex, education level, country of birth, and family/household composition will be gathered using a standard survey. Clinical data, including the duration of diabetes and current diabetes medications, will also be collected to describe the sample. Dietary intake will be assessed by a survey with short dietary intake questions. The survey includes five multiple-choice questions regarding the consumption of vegetables and fruits and 12 questions asking about the consumption of wholemeal/whole grain bread, milk, water, and some discretionary items. The single fruit and vegetable questions are adapted from those used in Australia’s National Nutrition Survey (1995), which were shown to discriminate between groups with different vegetable intakes assessed by a 24-h recall. The self-report Food Frequency questions are based on those used in the ShopSmart study [58]. Additional questions regarding the consumption of starchy vegetables and legumes were also designed and added to the survey to capture important outcomes for this T2D target group. Dietary self-efficacy and barriers as key predictors of dietary changes will be assessed by established self-report survey items, including 27 statements with Likert scale responses [59,60].

Feasibility and engagement will be evaluated by the number of participants recruited/retained, numbers accessing the website, logins and duration of website use, pages engaged with, and numbers receiving/reading the text messages [61]. For this purpose, web tagging is applied to the EatSmart website to record usage systematically. Tagging logs user interaction with particular program features and can provide real-time and historical statistics on engagement [62]. Website analytics allow us to identify whether portal login and usage are continuous, declining, or fluctuating during the intervention and follow-up period. In this intervention, we will consider adherence to the program to be confirmed by at least 66% of participants completing the study. We will also assess the number of website modules visited, as well as text messages read. While there is no set definition of feasibility in the literature for this context, we would consider the approach feasible if participants engage with the majority of materials (ie, on average more than 50% of website pages and text messages). However, we will also explore reasons for lower engagement levels through our semistructured interview to improve the materials for future larger studies. Program appeal, usability, and participants’ satisfaction will be assessed by self-report survey questions developed for this study. These include three statements with Likert scale responses and 11 open-ended qualitative questions about using the educational materials, useful features of the website and the messages, and liked and disliked aspects of the EatSmart program.

Participants will complete all self-report surveys, either web-based or telephone-assisted, at baseline and again at 3 and 9 months. The data capture and management tool REDCap (Research Electronic Data Capture) [63] will be used to collect data. Data will be entered automatically by the participant, via an individualized hyperlink or manually (by the research fellow), into REDCap. The data is stored, housed, and backed up within Deakin University in a database that can only be accessed via three layers of security (university firewalls, institute limitations, and project manager login).

Data on program appeal, usability, and participant satisfaction will be supplemented by semistructured interviews at 6 months post intervention to explore longer term maintenance of any behavioral or attitudinal changes.

At 9 months post baseline, participants will be invited by telephone, text message, or email to complete a one-on-one semistructured interview of up to 40 minutes via telephone. Recruitment for interviews will be successive until no new themes appear (thematic saturation). However, based on other qualitative reports in the same area [64-66], we do not expect to recruit fewer than 25 participants. Interview methods are useful for gaining specific information while allowing participants to freely share their viewpoints [62]. These interviews will be conducted to understand participants’ experiences of the EatSmart program and answer questions about whether, how, and why the intervention modules created long-lasting behavioral changes. The researcher will use a discussion guide of open-ended questions, and the interview will be audio recorded to ensure all verbal data are captured. The researcher will also write notes after the interview to document related nonverbal data. Participants will answer questions about the various parts of the website, text messages, and content presented within the intervention, their perceived capability for action within their personal situation, and any subsequent healthy eating-related changes.

These interviews will add to the program feedback from the surveys and create a base for understanding the characteristics of patients for whom these healthy changes occur and last. These data will provide us with insight into contextual factors that promote engagement.

At the end of the intervention, we will also conduct one-on-one interviews with up to ten health care providers involved with diabetes care (including endocrinologists, diabetes nurses, and diabetes nurse educators) at Western Health diabetes clinics. These interviews aim to understand providers’ views about successful or unsuccessful elements of EatSmart as a technology-delivered intervention, concerns or barriers regarding using these kinds of interventions, and feedback from their interactions with patients about the intervention’s content, impact, or observed benefits. The providers will be invited by email or face-to-face invitations to participate in a confidential semistructured interview of up to 30 min length. Interviews will be conducted at the health care practice or via telephone, as convenient for the provider. The interviews will begin by reviewing the website’s modules and text messages to guide the discussion. An open-ended discussion guide, audio recording, and note-taking of every session will be used to capture data. These data will complement the participant interviews to refine and develop the program for future studies.

### Statistical Analysis

Statistical analyses of quantitative and qualitative data will be conducted using Stata version 16 [67] and NVivo qualitative data analysis software (QSR International, version 10), respectively. Formal power calculations have not been conducted as this is a pilot study focusing on feasibility, and all of the comparisons and effects will be considered exploratory, regardless of the significance level found. However, the study will generate data on eating behaviors to inform power calculations for a larger subsequent trial.

Participants’ baseline and sociodemographic characteristics will be summarized using descriptive statistics. To understand the potential limitations of this intervention’s generalizability, characteristics of participants who prematurely exit the program will be compared with those who complete the program [68]. Potential intervention effects on dietary intake, self-efficacy, and perceived barriers will be assessed quantitatively by comparing pre- and postintervention and follow-up survey measures and analyzed using mixed models. Program feasibility measures will be analyzed as proportions, means, and frequencies. The frequency of participant logins and the number of visits to each page of the EatSmart website during the study and follow-up period will be extracted by web server logs and analyzed using descriptive analytical statistics. These analytical data will be used to describe participants’ engagement with the program generally and with specific modules during the intervention and follow-up period. Measures of program appeal and usability will be analyzed using descriptive analytical techniques (frequencies, proportions). Descriptive statistics will be derived from the survey responses, such as the reported extent of engagement with different website modules, the number of modules visited, the number of text messages read, and the perceived impact of the EatSmart intervention.

Furthermore, a thematic analysis will be employed for open-ended surveys to identify key program likes and dislikes. For qualitative measures from interviews, audiotapes of the interviews will be transcribed verbatim and de-identified. Transcripts will be imported to NVivo software to enable coding of the interview data and expedite the organization of codes into themes and subthemes. Two researchers will do line-by-line coding on three initial transcripts and then meet to discuss the development of preliminary conceptual themes and subthemes. These initial conceptual themes will then be applied to subsequent transcripts. Open discussions within the research team will resolve discrepancies in data interpretation. The coding process will continue until saturation, such that no further codes or categories can be found in the data. Thematic analysis will be used to describe why and among whom the program works best [69]. Qualitative results will be reported according to consolidated criteria for reporting qualitative research (COREQ) [70]. Incorporation of quantitative and qualitative analyses will thus happen at multiple stages in the evaluation of the program. Results from our different data sources will be combined and then interpreted to improve the validity of our conclusions.

## Results

The EatSmart website has been developed, tailored to the needs of people of lower SEP with T2D, and all the modules viewed by participants. Recruitment began in October 2019 and finished in February 2020. Data collection will continue through October 2020. Data analysis and cleaning will be conducted after data collection is complete. We anticipate reporting results in December 2020 by submitting professional publications in peer-reviewed journals and conference presentations.

## Discussion

As with many chronic diseases, a disproportionately high burden of diabetes and its associated complications is shouldered by those who are socioeconomically disadvantaged. This target group is often neglected in research trials as they are generally considered too hard to reach and impact [71]. EatSmart is one of the first international studies to deliver theoretically grounded, evidence-based support digitally to enable disadvantaged people with T2D to strengthen skills necessary to eat healthily on a budget, as a key part of diabetes self-management. EatSmart offers a potentially low-cost approach with high reach, with the only cost to participants involving minimal data use to access the website from their existing internet service.

Several approaches have been employed to increase the efficacy of EatSmart. Firstly, in all stages of the website’s design, development, and content, the end users’ particular needs and perspectives have been evaluated and considered. Secondly, a behavioral support reminder system in the form of regular text messages helps promote continued engagement and address the problem of high attrition rates, a problem common to eHealth studies [72]. Furthermore, the intervention framework is theoretically grounded in Bandura’s Social Cognitive Theory [47], emphasizing the importance of factors within the cognitive, socioenvironmental, and behavioral domains and their interactions. Key constructs of Social Cognitive Theory are goals (plans to act that can be deemed as intentions to perform the behavior), self-efficacy (peoples’ judgment of their ability to perform a behavior), and outcome expectations (views about the outcomes that are possible to result from a specific behavior).

EatSmart targets nutritional knowledge gaps and misinformation, promotes positive mindsets toward healthy eating (ie, self-efficacy), and facilitates changes in eating behaviors to increase self-efficacy and behavior change. Active learning, guided practice, reinforcement, and modeling are key features of Social Cognitive Theory contained within EatSmart. Some of the strategies incorporated into the design of EatSmart included goal setting and problem solving for building self-management skills, providing hands-on skill-building activities (eg, cooking activities utilizing healthy ingredients and cooking methods), and direct instruction and modeling by the intervention nutritionist (in videos). Modeling is an important influence, providing individuals with skills and strategies to adopt and maintain behaviors in different situations. The video components of the EatSmart website incorporate modeling, with actors (research team members) providing support in real-world environments such as the supermarket and the kitchen at home. The intervention also draws on empirical evidence of the key determinants of eating behaviors, including our past work suggesting the significance of addressing nutrition knowledge, food planning and budgeting and preparation skills, perceived and actual food costs, and other barriers [11]. Finally, the mixed methods design and the inclusion of in-depth qualitative interviews can provide deep insight into the challenges and promises of employing eHealth healthy eating interventions for disadvantaged people with T2D.

The results of this pilot study will be interpreted with consideration of the following limitations. The study relies on self-reported measures, which may be subject to recall and social desirability bias. The lack of a control group is another limitation, although this is an appropriate design for a pilot study in a relatively new area, with feasibility and exploratory focus. Furthermore, while clinicians and 12 randomly selected patients with T2D were involved in reviewing and providing input to a draft website, there could have been greater involvement from these groups throughout the development of the intervention. However, as it is a pilot study, the feedback survey and interviews will be used to further inform the development of future interventions. Finally, EatSmart is currently only presented in English, which is essential to evaluate the feasibility of this initial pilot trial. Translation to other languages can be a future priority to reach other underserved communities at higher risk of health disparities.

The EatSmart intervention study results will make a valuable contribution to the evidence base on diabetes self-management in a high-risk population. This project will inform scalable public health programs to promote healthy eating and diabetes self-management as an inexpensive adjunct to clinical care among vulnerable populations and contribute new knowledge to digitally delivered health research in Australia and internationally.

## Appendix

Multimedia Appendix 1 Participant Information and Consent Form.

Multimedia Appendix 2 Participant Information and Consent Form (Follow up interview).

Multimedia Appendix 3 HCP Information and Consent Form.

Multimedia Appendix 4 Text messages provided in the EatSmart program.

Multimedia Appendix 5 Study measures and assessment tools across time points.

## Abbreviations

SEP: socioeconomic position
T2D: type 2 diabetes
