# Supplementary material for: EatSmart, a Web-Based and Mobile Healthy Eating Intervention for Disadvantaged People With Type 2 Diabetes: Protocol for a Pilot Mixed Methods Intervention Study
Source: JMIR Res Protoc. 2020 Nov 6;9(11):e19488. doi: 10.2196/19488 (PMC7679211; doi:10.2196/19488)
Supplement: Multimedia Appendix 1 [file resprot_v9i11e19488_app1.doc]

# Western Health Low Risk Human Research Ethics Panel

###### Participant Information and Consent Form

Participant Information and Consent Form

Version: 2 Dated: 23.01.2019
Site: *Sunshine Hospital*

Full Project Title: A novel approach for supporting healthy eating on a budget for people with Type 2 diabetes

**Principal Researcher:** Professor Kylie Ball

**Co-principal investigator:** Associate Professor Peter (Shane) Hamblin

**Associate Researcher(s):** Dr Rachelle Opie, Professor Ralph Maddison, Professor Bodil Rasmussen, Dr Ashley Ng, Professor David Crawford, Ms Nazgol Karimi, Dr. Stella O’Connell, Ms Cheryl Steele

This Participant Information and Consent Form is 6 pages long. Please make sure you have all the pages.

1. Your Consent

You are invited to take part in this research project as you live with type 2 diabetes. Your involvement will help us learn about the kinds of programs that might help people with Type 2 diabetes to eat healthily on a budget.

This Participant Information contains detailed information about the research project. Its purpose is to explain to you as openly and clearly as possible all the procedures involved in this project before you decide whether or not to take part in it.

Please read this Participant Information carefully. Feel free to ask questions about any information in the document. You may also wish to discuss the project with a relative or friend or your local health worker. Feel free to do this.

Once you understand what the project is about and if you agree to take part in it, you will be asked to sign the Consent Form. By signing the Consent Form, you indicate that you understand the information and that you give your consent to participate in the research project.

You will be given a copy of the Participant Information and Consent Form to keep as a record.

2. Purpose and Background

The purpose of this project is to test if a program focused on healthy eating on a budget program is appealing and effective for socioeconomically disadvantaged adults with Type 2 diabetes.

A total of 60 people will be recruited.

Healthy eating, including lots of vegetables, fruit, wholegrain cereals and limited amounts of processed foods, is an important part of managing Type 2 diabetes. However, many people find healthy eating hard, for a range of reasons. These can include not knowing or having the skills to choose and prepare healthy foods; or difficulties buying healthy foods on a budget. This can be particularly challenging for people who are on low incomes, since there are many pressures on the household budget.

We have developed a healthy eating on a budget program, which is specifically designed for people with Type 2 diabetes who have a low income.

We would like to test this program to see if it is helpful and appealing. You are invited to participate in this research project because you are currently living with Type 2 diabetes. Your involvement will help us learn about the kinds of programs that might help people with Type 2 diabetes to eat healthily on a budget. It is hoped that this program will help people with Type 2 diabetes to improve their eating habits as well as better self-manage their condition and health. The results of this research will help us design and refine future healthy eating programs to help others. The results may also be used to help researcher Nazgol Karimi to obtain a PhD.

3. Procedures

Participation in this project will involve completing a survey before the program starts, and another survey at the end of the program (at 12 weeks). The survey and introduction to the program will take about one hour. You can complete the survey either on your own, or with the help of one of our researchers (in person or by telephone).

The survey asks about your diabetes history, your eating patterns, and your attitudes about eating. The program runs for 12 weeks, and it is designed to provide useful information, skills and support for heathy eating. It is delivered by internet (website) and mobile telephone Short Message System (SMS). You can do the program in your own time (for example from home), and we expect it will take up to two hours per week at times convenient to you. As part of the program you will receive three text messages per week from us, providing support to help you with the program.

At the end of the 12 week program, the final survey will take around 30 minutes to complete. You will receive a $50 shopping voucher after completing the final survey to thank you and as compensation for your time.

4. Possible Benefits

Possible benefits include a healthier dietary intake, and increased nutrition knowledge and skills to help you eat more healthily. We cannot guarantee or promise that you will receive any benefits from this project.

5. Possible Risks

Possible risks, side effects and discomforts posed by the intervention are unlikely. The program is designed to supplement your usual care. You are encouraged to continue your usual treatment regime and speak with your GP or treating professional about any health concerns. There may be unforeseen or unknown risks.

6. Alternatives to Participation

You do not have to participate in this research project to receive any medical care you may require. Your ongoing care at Western Health will not be affected in any way if you decide to participate or not participate.

7. Privacy, Confidentiality and Disclosure of Information

All data generated will be saved in a locked filing cabinet or as computer files which will be password protected and only members of the research team will be able to access these files. Data will be stored in a re-identifiable form (e.g. de-identified with a unique ID to allow for matching of your data before and after the program). Data will be destroyed five years after the study results are published.

Any information obtained in connection with this project and that can identify you will remain confidential. It will only be disclosed with your permission, except as required by law. If you give us your permission by signing the Consent Form, we plan to share the findings with key stakeholders and publish the results in high quality peer-reviewed journals. In any publication, information will be provided in such a way that you cannot be identified. Only group data from this study will be reported in publications.

In accordance with relevant Australian and/or Victorian privacy and other relevant laws you have the right to access the information collected and stored by the researchers about you. You also have the right to request that any information with which you disagree be corrected. Please contact one of the researchers named below if you would like to access your information.

8. New Information Arising During the Project

During the research project, new information about the risks and benefits of the project may become known to the researchers. If this occurs, you will be told about this new information. This new information may mean that you can no longer participate in this research. If this occurs, the person(s) supervising the research will stop your participation. In all cases, you will be offered all available care to suit your needs and medical condition.

9. Results of Project

Upon completion of this research, a summary of the results will be available to you if you request this from the researchers, by email at [ipan@deakin.edu.au](mailto:ipan@deakin.edu.au).

The data will contain no identifying personal information, and only group results and anonymous quotes will be presented. The results of the study will be reported in conference presentations, peer-reviewed publications, and a summary of findings will be shared with key stakeholders.

11. Further Information or Any Problems

If you require further information or if you have any problems concerning this project, you can contact the study coordinator Dr Stella O’Connell (ph 9244 6135), the Principal Investigator at Western Health Associate Professor Shane Hamblin (ph 8345 0860) or the researchers at Deakin University. The researchers responsible for this project are Professor Kylie Ball (ph 9251 7310), Dr Rachelle Opie (ph 9246 8381), Professor Ralph Maddison, Professor Bodil Rasmussen, Dr Ashley Ng, Professor David Crawford, Ms Nazgol Karimi, and Ms Cheryl Steele.

12. Other Issues

If you have any complaints about any aspect of the project, the way it is being conducted or any questions about your rights as a research participant, then you may contact:

| Position: | Manager, Western Health Office for Research |
| --- | --- |
| Telephone: | (03) 8395 8073 |
| Email: | ethics@wh.org.au |

(You will need to tell the Manager the name of one of the researchers given in section 11 above.)

13. Participation is Voluntary

Participation in any research project is voluntary. If you do not wish to take part you are not obliged to. If you decide to take part and later change your mind, you are free to withdraw from the project at any stage.

Your decision whether to take part or not to take part, or to take part and then withdraw, will not affect your routine treatment, your relationship with those treating you or your relationship with Deakin University and Western Health.

Before you make your decision, a member of the research team will be available to answer any questions you have about the research project. You can ask for any information you want. Sign the Consent Form only after you have had a chance to ask your questions and have received satisfactory answers.

If you decide to withdraw from this project, please notify a member of the research team before you withdraw. This notice will allow that person or the research supervisor to inform you if there are any health risks or special requirements linked to withdrawing.

14. Ethical Guidelines

This project will be carried out according to the *National Statement on Ethical Conduct in Human Research* (2007) produced by the National Health and Medical Research Council of Australia. This statement has been developed to protect the interests of people who agree to participate in human research studies.

The ethical aspects of this research project have been approved by the Western Health Low Risk Human Research Ethics Panel.

15. Reimbursement for your costs

You will not be paid for your participation in this project.

However, you will receive a $50 shopping voucher after the completion of the final survey to thank you for your time.

**
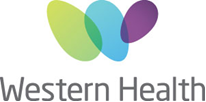
**

**16.** Consent Form

| Site: Sunshine Hospital  **Project title:** A novel approach for supporting healthy eating on a budget among people with Type 2 diabetes |
| --- |

I have read, and I understand the Participant Information.

I freely agree to participate in this project according to the conditions in the Participant Information.

I will be given a copy of the Participant Information and Consent Form to keep

The researcher has agreed not to reveal my identity and personal details if information about this project is published or presented in any public form.

Participant’s Name (printed) ……………………………………………………

Signature………………………………… Date

Name of Witness to Participant’s Signature (printed) …………………………………

Signature………………………………… Date

Declaration by researcher*: I have given a verbal explanation of the research project, its procedures and risks and I believe that the participant has understood that explanation.

Researcher’s Name (printed) ……………………………………………………

Signature………………………………… Date

* A senior member of the research team must provide the explanation and provision of information concerning the research project.

*Note:* All parties signing the Consent Form must date their own signature.


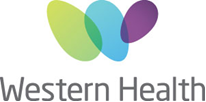


**REVOCATION OF CONSENT FORM**

Revocation of Consent Form

| Full Project Title: A novel approach for supporting healthy eating on a budget among people with Type 2 diabetes |
| --- |

I hereby wish to WITHDRAW my consent to participate in the research proposal described above and understand that such withdrawal WILL NOT jeopardise any treatment or my relationship with Deakin University and Western Health.

Participant’s Name (printed) ……………………………………………………

Signature………………………………… Date
