# Supplementary material for: EatSmart, a Web-Based and Mobile Healthy Eating Intervention for Disadvantaged People With Type 2 Diabetes: Protocol for a Pilot Mixed Methods Intervention Study
Source: JMIR Res Protoc. 2020 Nov 6;9(11):e19488. doi: 10.2196/19488 (PMC7679211; doi:10.2196/19488)
Supplement: Multimedia Appendix 2 [file resprot_v9i11e19488_app2.doc]

# Western Health Low Risk Human Research Ethics Panel

###### Participant Information and Consent Form – Patients

Participant Information and Consent Form

Version: 1 Dated: 23.06.2020
Site: *Sunshine Hospital*

Full Project Title: A novel approach for supporting healthy eating on a budget for people with Type 2 diabetes

**Principal Researcher:** Professor Kylie Ball

**Co-principal investigator:** Associate Professor Peter (Shane) Hamblin

**Associate Researcher(s):** Dr Rachelle Opie, Professor David Crawford, Ms Nazgol Karimi, Ms Cheryl Steele

This Participant Information and Consent Form is 7 pages long. Please make sure you have all the pages.

1. Your Consent

You are being invited to continue to take part in a research study about “EatSmart, a novel approach for supporting healthy eating on a budget for people with Type 2 diabetes”.

Previously, you participated in the first phase of this healthy eating program, and we are very grateful for your contribution. It has now been 6 months since you took part in the program, so we are interested in hearing about any further changes you have experienced with regards to your eating habits, as well as any valuable ideas you may have about the program itself.

Your involvement will help us to learn about your likes and dislikes of EatSmart and the characteristics and specifications of the programs that might have helped you to improve your healthy eating behaviours. Based on this knowledge, we can refine and develop future public health programs which can support you and other people with diabetes.

This Participant Information contains detailed information about the research project. Its purpose is to explain to you as openly and clearly as possible all the procedures involved in this project before you decide whether or not to take part in it.

Please read this Participant Information carefully. Take as much time as you need to make your decision. You can ask the researcher, Nazgol Karimi, to explain anything that you do not understand and make sure that all your questions have been answered before agreeing to participate in this study. Feel free to talk about this study with anyone you wish. Ongoing participation in this study is completely voluntary and you can withdraw at any time.

Once you understand what the project is about and if you agree to take part in it, you will be asked to sign the Consent Form. By signing the Consent Form, you indicate that you understand the information and that you give your consent to participate in the research project.

You will be given a copy of the Participant Information and Consent Form to keep as a record.

2. Background and Purpose

Healthy eating, including a variety of vegetables, fruits, wholegrain cereals and limited amounts of processed foods, is an important part of managing Type 2 diabetes. However, many people find healthy eating challenging, for a range of reasons. These can include not knowing or having the skills to choose and prepare healthy foods; or difficulties buying healthy foods on a budget. This can be particularly challenging for people who are on low incomes since there are many pressures on the household budget. The program you were part of, EatSmart, was a web-based and phone delivered healthy eating on a budget program, which was specifically designed for people with Type 2 diabetes who have a low income. The purpose of continuing this project is to find out whether participating in this program can lead to long-lasting changes in healthy eating behaviours 6 months post-program. The results of this research will help us design and refine future healthy eating programs. The results may also be used to help researcher Nazgol Karimi to obtain a PhD.

3. Procedures

Online survey:

If you agree to continue to take part in the study, you will be asked to complete an online follow-up survey (the same diet survey you previously completed, about your diabetes history, eating patterns, and your attitudes about eating.). The survey will take approximately 20 minutes to complete. You can complete the survey either in your own time or with the help of one of our researchers (by telephone). In recognition of your contribution, you will receive a $20 gift voucher after completing this survey.

Telephone interview:

If you also indicate your willingness to take part in a separate telephone interview, we will contact you for a follow-up interview. During the interview, we will ask you about your views and experiences of EatSmart. The interviews will take place at a date and time convenient to you and will take approximately 40 minutes. In recognition of your contribution, you will receive a $20 gift voucher after this telephone interview.

Note: If you are not comfortable in completing the survey online, we can help you to do that by phone, at the same time with the interview. In that case the procedure (Survey and interview) will last 60 minutes.

4. Possible Benefits

Your information may help the development of more appropriate tools and approaches to support you and other people with type 2 diabetes to follow healthier eating patterns.

5. Possible Risks

We do not believe that this study poses any risks and is unlikely to cause you any harm. However, if you experience any discomfort or distress when completing any of the survey questions or interviews, you have the right not to answer the question and you may also withdraw from the study at any time.

6. Alternatives to Participation

You do not have to participate in this research project to receive any medical care you may require. Your ongoing care at Western Health will not be affected in any way if you decide to participate or not participate.

7. Privacy, Confidentiality and Disclosure of Information

All information obtained during the study will be held in strict confidence. All data generated will be saved in a locked filing cabinet or as computer files which will be password protected and only members of the research team will be able to access these files. Data will be stored in a re-identifiable form (e.g. de-identified with a unique ID to allow for matching of your data before and after the program). Data will be destroyed five years after the study results are published.

Any information obtained in connection with this project and that can identify you will remain confidential. It will only be disclosed with your permission, except as required by law. If you give us your permission by signing the Consent Form, we plan to share the de-identified findings with key stakeholders and publish the results in high-quality peer-reviewed journals. In any publication, the information will be provided in such a way that you cannot be identified. Only group data from this study will be reported in publications.

In accordance with relevant Australian and/or Victorian privacy and other relevant laws you have the right to access the information collected and stored by the researchers about you. You also have the right to request that any information with which you disagree be corrected. Please contact one of the researchers named below if you would like to access your information.

8. New Information Arising During the Project

During the research project, new information about the risks and benefits of the project may become known to the researchers. If this occurs, you will be told about this new information. This new information may mean that you can no longer participate in this research. If this occurs, the person(s) supervising the research will stop your participation. In all cases, you will be offered all available care to suit your needs and medical condition.

9. Results of Project

Upon completion of this research, a summary of the results will be available to you if you request this from the researchers, by email at [ipan@deakin.edu.au](mailto:ipan@deakin.edu.au).

The data will contain no identifying personal information, and only group results and anonymous quotes will be presented. The results of the study will be reported in conference presentations, peer-reviewed publications, and a summary of findings will be shared with key stakeholders.

11. Further Information or Any Problems

If you require further information or if you have any problems concerning this project, you can contact the study coordinator Ms Nazgol Karimi (ph 410825300), the Principal Investigator at Western Health Associate Professor Shane Hamblin (ph 8345 0860) or the researchers at Deakin University. The researchers responsible for this project are Professor Kylie Ball (ph 9251 7310, email: kylie.ball@deakin.edu.au), Dr Rachelle Opie (ph 9246 8381, email: rachelle.opie@deakin.edu.au), Professor David Crawford, and Ms Cheryl Steele.

12. Other Issues

If you have any complaints about any aspect of the project, the way it is being conducted or any questions about your rights as a research participant, then you may contact:

| Position: | Manager, Western Health Office for Research |
| --- | --- |
| Telephone: | (03) 8395 8073 |
| Email: | ethics@wh.org.au |

(You will need to tell the Manager the name of one of the researchers given in section 11 above.)

13. Participation is Voluntary

Participation in any research project is voluntary. If you do not wish to take part you are not obliged to. If you decide to take part and later change your mind, you are free to withdraw from the project at any stage.

Your decision whether to take part or not to take part, or to take part and then withdraw, will not affect your routine treatment, your relationship with those treating you or your relationship with Deakin University and Western Health.

Before you make your decision, a member of the research team will be available to answer any questions you have about the research project. You can ask for any information you want. Sign the Consent Form only after you have had a chance to ask your questions and have received satisfactory answers.

If you decide to withdraw from this project, please notify a member of the research team before you withdraw. This notice will allow that person or the research supervisor to inform you if there are any health risks or special requirements linked to withdrawing.

14. Ethical Guidelines

This project will be carried out according to the *National Statement on Ethical Conduct in Human Research* (2007) produced by the National Health and Medical Research Council of Australia. This statement has been developed to protect the interests of people who agree to participate in human research studies.

The ethical aspects of this research project have been approved by the Western Health Low Risk Human Research Ethics Panel.

15. Reimbursement for your costs

You will not be paid for your participation in this project.

However, you will receive a $20 shopping voucher after completing the online survey and a $20 shopping voucher after completing the telephone interview to thank you for your time.


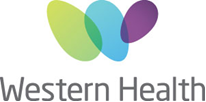


**16.** Consent Form

| Site: Sunshine Hospital  **Project title:** A novel approach for supporting healthy eating on a budget among people with Type 2 diabetes |
| --- |

I have read, and I understand the Participant Information.

I freely agree to participate in this project according to the conditions in the Participant Information.

I will be given a copy of the Participant Information and Consent Form to keep

The researcher has agreed not to reveal my identity and personal details if information about this project is published or presented in any public form.

Participant’s Name (printed) ……………………………………………………

Signature………………………………… Date

Name of Witness to Participant’s Signature (printed) …………………………………

Signature………………………………… Date

Declaration by researcher*: I have given a verbal explanation of the research project, its procedures and risks and I believe that the participant has understood that explanation.

Researcher’s Name (printed) ……………………………………………………

Signature………………………………… Date

* A senior member of the research team must provide the explanation and provision of information concerning the research project.

*Note:* All parties signing the Consent Form must date their own signature.

*
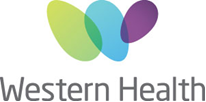
*

**REVOCATION OF CONSENT FORM**

Revocation of Consent Form

| Full Project Title: A novel approach for supporting healthy eating on a budget among people with Type 2 diabetes |
| --- |

I hereby wish to WITHDRAW my consent to participate in the research proposal described above and understand that such withdrawal WILL NOT jeopardise any treatment or my relationship with Deakin University and Western Health.

Participant’s Name (printed) ……………………………………………………

Signature………………………………… Date
