# Supplementary material for: EatSmart, a Web-Based and Mobile Healthy Eating Intervention for Disadvantaged People With Type 2 Diabetes: Protocol for a Pilot Mixed Methods Intervention Study
Source: JMIR Res Protoc. 2020 Nov 6;9(11):e19488. doi: 10.2196/19488 (PMC7679211; doi:10.2196/19488)
Supplement: Multimedia Appendix 3 [file resprot_v9i11e19488_app3.doc]

# Western Health Low Risk Human Research Ethics Panel

###### Participant Information and Consent Form

###### Healthcare Providers

Participant Information and Consent Form

Version: 1 Dated: 23.06.2020
Site: *Sunshine Hospital*

Full Project Title: A novel approach for supporting healthy eating on a budget for people with Type 2 diabetes

**Principal Researcher:** Professor Kylie Ball

**Co-principal investigator:** Associate Professor Peter (Shane) Hamblin

**Associate Researcher(s):** Dr Rachelle Opie, Professor Ralph Maddison, Professor Bodil Rasmussen, Dr Ashley Ng, Professor David Crawford, Ms Nazgol Karimi, Dr. Stella O’Connell, Ms Cheryl Steele

This Participant Information and Consent Form is 6 pages long. Please make sure you have all the pages.

1. Your Consent

You are being invited to take part in an interview about a research project, entitled “EatSmart, a novel approach for supporting healthy eating on a budget for people with Type 2 diabetes (T2D)”.

This project tested an evidence-based, theoretically grounded, web-based and mobile intervention, designed to help people with T2D on a low income to eat healthy foods on a budget, in order to better self-manage their condition. It is designed with input from people with T2D, researchers in dietetics, behaviour change, technology-delivered programs, diabetes care and clinicians (Endocrinologists, Diabetes Nurse Educator).

Your involvement in this interview will help us to identify the successful and unsuccessful elements of EatSmart as a digital-delivered intervention from your perspective, and also understand your views regarding using these kinds of interventions as an inexpensive adjunct to usual clinical care.

This Participant Information contains detailed information about the research project. Its purpose is to explain to you as openly and clearly as possible all the procedures involved in this project before you decide whether or not to take part in it.

Please read this Participant Information carefully. Feel free to ask questions about any information in the document. Once you understand what the project is about and if you agree to take part in it, you will be asked to sign the Consent Form. By signing the Consent Form, you indicate that you understand the information and that you give your consent to participate in the research project.

You will be given a copy of the Participant Information and Consent Form to keep as a record.

2. Background and Purpose

Healthy eating, including a variety of vegetables, fruits, wholegrain cereals and limited amounts of processed foods, is an important part of managing Type 2 diabetes. However, many people find healthy eating challenging, for a range of reasons. These can include not knowing or having the skills to choose and prepare healthy foods; or difficulties buying healthy foods on a budget. This can be particularly challenging for people who are on low incomes since there are many pressures on the household budget. We developed a healthy eating on a budget program, which was specifically designed for disadvantaged people with T2D. This program, funded by a 2019 Diabetes Australia Research Program Grant, aims to test the appeal, feasibility and potential effects on eating behaviours and related skills and attitudes of a scalable, evidence-based, web-based and mobile delivered healthy eating program for socioeconomically disadvantaged people with T2D. Besides, we want to explore whether participating in this program can be associated with sustained changes in healthy eating behaviours 6 months after the completion of the program.

A total of sixty socioeconomically disadvantaged people with T2D aged 18 to 75 years were recruited. Participants provided with log-in access to the EatSmart web-program, which includes six progressive skill-based modules covering healthy eating planning; smart food shopping; time-saving meal strategies; cooking; modifying recipes; and a final reinforcement and summary module. Over the three-month intervention, participants also received three text messages weekly of encouragement to review goals and continue to engage with different components of the EatSmart web program.

You are invited to be interviewed about this research project because as a health care provider who is involved with diabetes care, you have invaluable knowledge about the special needs of people with T2D from lower socio-economic status. This interview aims to understand your views about successful or unsuccessful elements of EatSmart, concerns or barriers regarding the use of these kinds of interventions, and if you had any patients take part, any feedback you may have had about your interactions with patients and the long-term impact or observed benefits. Your contribution will help us to refine and develop the program for future studies. The results will also be used to help researcher Nazgol Karimi to obtain a PhD.

3. Procedures

Take part in a telephone (Zoom) interview:

In this interview we would like to hear your opinions about successful or unsuccessful elements of EatSmart as a digital-delivered intervention and if you had any patients take part, any feedback you may have had about your interactions with patients and the long-term impact or observed benefits. The interview will take around 30 minutes and it will be audio recorded to ensure all verbal data is captured. The audiotape of your interview will be transcribed verbatim and de-identified.

4. Possible Benefits

We cannot guarantee or promise that you will receive any direct benefits from this interview. But your contribution will help us to refine and develop future scalable digital public health programs which can serve as an inexpensive adjunct to clinical care among populations from lower socio-economic background.

5. Possible Risks

Possible risks and discomforts posed by the interview are unlikely.

6. Alternatives to Participation

You do not have to participate in this interview, but we would very much like to know your perspectives about this program. In case you don’t have time for the telephone interview, we can email the interview questions so you can answer them at a time convenient to you.

7. Privacy, Confidentiality and Disclosure of Information

All data generated will be saved in a locked filing cabinet or as computer files which will be password protected and only members of the research team will be able to access these files. Data will be stored in a re-identifiable form (e.g. de-identified with a unique ID to allow for matching of your data before and after the program). Data will be destroyed five years after the study results are published.

Any information obtained in connection with this project and that can identify you will remain confidential. It will only be disclosed with your permission, except as required by law. If you give us your permission by signing the Consent Form, we plan to share the findings with key stakeholders and publish the results in high quality peer-reviewed journals. In any publication, information will be provided in such a way that you cannot be identified. Only group data from this study will be reported in publications.

In accordance with relevant Australian and/or Victorian privacy and other relevant laws you have the right to access the information collected and stored by the researchers about you. You also have the right to request that any information with which you disagree be corrected. Please contact one of the researchers named below if you would like to access your information.

8. New Information Arising During the Project

During the research project, new information about the risks and benefits of the project may become known to the researchers. If this occurs, you will be told about this new information. This new information may mean that you can no longer participate in this research. If this occurs, the person(s) supervising the research will stop your participation. In all cases, you will be offered all available care to suit your needs and medical condition.

9. Results of Project

Upon completion of this research, a summary of the results will be available to you if you request this from the researchers, by email at [ipan@deakin.edu.au](mailto:ipan@deakin.edu.au).

The data will contain no identifying personal information, and only group results and anonymous quotes will be presented. The results of the study will be reported in conference presentations, peer-reviewed publications, and a summary of findings will be shared with key stakeholders.

11. Further Information or Any Problems

If you require further information or if you have any problems concerning this project, you can contact the study coordinator Ms Nazgol Karimi (ph 410825300), the Principal Investigator at Western Health Associate Professor Shane Hamblin (ph 8345 0860) or the researchers at Deakin University. The researchers responsible for this project are Professor Kylie Ball (ph 9251 7310, email: kylie.ball@deakin.edu.au), Dr Rachelle Opie (ph 9246 8381, email: rachelle.opie@deakin.edu.au), Professor David Crawford, and Ms Cheryl Steele.

12. Other Issues

If you have any complaints about any aspect of the project, the way it is being conducted or any questions about your rights as a research participant, then you may contact:

| Position: | Manager, Western Health Office for Research |
| --- | --- |
| Telephone: | (03) 8395 8073 |
| Email: | ethics@wh.org.au |

(You will need to tell the Manager the name of one of the researchers given in section 11 above.)

13. Participation is Voluntary

Your participation in this interview is completely voluntary. This means that you do not have to participate in this interview unless you want to. And you can also refuse to answer any questions or terminate the interview at any time.

14. Ethical Guidelines

This project will be carried out according to the *National Statement on Ethical Conduct in Human Research* (2007) produced by the National Health and Medical Research Council of Australia. This statement has been developed to protect the interests of people who agree to participate in human research studies.

The ethical aspects of this research project have been approved by the Western Health Low Risk Human Research Ethics Panel.

15. Reimbursement for your costs

You will not be paid for your participation in this project.

**
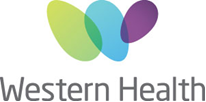
**

**16.** Consent Form

| Site: Sunshine Hospital  **Project title:** A novel approach for supporting healthy eating on a budget among people with Type 2 diabetes |
| --- |

I have read, and I understand the Participant Information.

I freely agree to participate in this project according to the conditions in the Participant Information.

I will be given a copy of the Participant Information and Consent Form to keep

The researcher has agreed not to reveal my identity and personal details if information about this project is published or presented in any public form.

Participant’s Name (printed) ……………………………………………………

Signature………………………………… Date

Name of Witness to Participant’s Signature (printed) …………………………………

Signature………………………………… Date

Declaration by researcher*: I have given a verbal explanation of the research project, its procedures and risks and I believe that the participant has understood that explanation.

Researcher’s Name (printed) ……………………………………………………

Signature………………………………… Date

* A senior member of the research team must provide the explanation and provision of information concerning the research project.

*Note:* All parties signing the Consent Form must date their own signature.


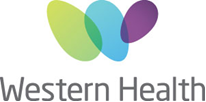


**REVOCATION OF CONSENT FORM**

Revocation of Consent Form

| Full Project Title: A novel approach for supporting healthy eating on a budget among people with Type 2 diabetes |
| --- |

I hereby wish to WITHDRAW my consent to participate in the research proposal described above and understand that such withdrawal WILL NOT jeopardize my relationship with Deakin University and Western Health.

Participant’s Name (printed) ……………………………………………………

Signature………………………………… Date
