# Supplementary material for: EatSmart, a Web-Based and Mobile Healthy Eating Intervention for Disadvantaged People With Type 2 Diabetes: Protocol for a Pilot Mixed Methods Intervention Study
Source: JMIR Res Protoc. 2020 Nov 6;9(11):e19488. doi: 10.2196/19488 (PMC7679211; doi:10.2196/19488)
Supplement: Multimedia Appendix 4 [file resprot_v9i11e19488_app4.doc]

Multimedia Appendix 4: Text messages provided in the EatSmart program.

| **EatSmart Module** | **Message** | **EatSmart Module** | **Message** |
| --- | --- | --- | --- |
|  |  |  |  |
| **1. Eating smart: how to start** | Hi *(Participant’s name)*, welcome to the EatSmart program! Over the next 12 weeks, we’ll focus on three key healthy eating habits: eating plenty of vegetables, two pieces of fruit and plenty of water each day. Healthy eating is important for diabetes management and keeping your blood sugars within target range. Get started by checking out our website <http://eatsmart.org.au/> | **4. Cooking smart** | Hi *(Participant’s name)*, Congratulations, You’re more than half-way through the EatSmart program! Welcome to Module 4 – Cooking Smart! Hopefully you are feeling more confident about shopping and eating Smart - now learn more about preparing healthy meals. Start with great ideas for preparing healthy snacks by watching Lena in her next video <http://eatsmart.org.au/>Enjoy! Stella. |
|  | Hi *(Participant’s name)*, Stella here from EatSmart. Setting a goal is one of the best ways to start healthier eating. If you haven’t already, think about setting one goal to help you with healthy eating this week, e.g. eat two extra serves of vegetables or some fruit with lunch or dinner. Writing your goals helps you stick to them, so record yours on our website <http://eatsmart.org.au/> |  | Hi *(Participant’s name)*, it’s Stella, *(Participant’s name)*. How are you going with your healthy eating goals? If you’re not meeting them, what are the key challenges, and how can you prepare for these? In Module 4, we suggest you set a goal to try a new way of cooking vegetables – maybe stir-frying, microwaving or steaming, which are all quick and nutritious. See the website for how! <http://eatsmart.org.au/> |
|  | Hi *(Participant’s name*), Eating 5 serves of vegetables and 2 serves of fruit each day is a great way to get all the healthy nutrients you need and help manage your diabetes. Plan how you’ll get extra serves to meet your goal: e.g. add a vegetable to your sandwich or dinner. Do you know how much one serve is, *(Participant’s name*)? Find out in Module 1, then try our fun quizzes! <http://eatsmart.org.au/> |  | Hi *(Participant’s name)*, Stella here. Each time you serve a meal, fill half of your plate with veggies and fruits. Now fill a quarter with wholegrain cereals like brown rice or pasta, and the last quarter with some protein food, like lean meat, beans or tofu. For 10 healthy ways to cook veggies and fruit see <https://www.fruitsandveggiesmorematters.org/top-10-healthy-ways-to-cook-fruits-and-vegetables>. |
|  | Hi *(Participant’s name)*, How did you go with your healthy eating goal for Module 1? If you achieved it, great! Reward yourself with an activity you enjoy, like reading a magazine, a chat with friends, or a walk in the park. This week set a slightly higher goal! If you didn’t meet your goal, don’t give up! Change can be hard, let’s try again now. <http://eatsmart.org.au/> |  | Hi *(Participant’s name)*, Hungry? Serve delicious home-made dips with carrot, celery or cucumber sticks; or a dessert of fruit with low-fat yoghurt. When friends or family visit, suggest they bring their favorite fruit or vegetable dish to share. Tasty pasta or Basmati rice with a tomato-based sauce and lots of added vegies makes great low-budget food. <http://eatsmart.org.au/> Stella |
|  | Hi *(Participant’s name)*, Drinking water is vital for every part of your body! How about making an extra goal to swap a sugary drink with tap water every day this week? As a change from plain water, try adding a squeeze of lemon or lime; mint leaves; sliced cucumber; or colorful berries or melon pieces. On festive occasions, enjoy special foods, but maybe eat a bit less of them or plan for small healthy meals around them. Stella <http://eatsmart.org.au/> |  | Hi *(Participant’s name)*, It’s Stella again. Remove, Reduce, Replace: these 3Rs are three simple steps for changing a recipe to a healthier one. Watch the Module 4 video which shows you how the 3Rs make small healthy changes in your cooking that add up to great tasty food that’s better for you. It’s often cheaper too! <http://eatsmart.org.au/> |
|  | Hi (Participant’s name), Different colored fruits and vegetables provide different vitamins and minerals, so 'eating a rainbow' is a great way to help get everything you need to stay healthy. How many colors do you eat? Aim to include red, orange, yellow, green, white and purple veggies in your next shop. Find out what’s in each color at http://www.nutritionaustralia.org/national/resource/eat-rainbow Enjoy! Stella |  | Hi *(Participant’s name)*, Need more tasty recipe ideas? Try our delicious Module 4 tomato recipes! <http://eatsmart.org.au/> Making your own food instead of buying it can save you a lot of money and you’ll know exactly what’s in it, too! Compare the cost of your take-away pizza to our colorful home-made version. Stella |
| **2. Eating smart on a budget** | Hi *(Participant’s name)*, welcome to EatSmart Module 2, just released: Eating Smart on a Budget! Healthy eating starts with planning and buying the right kinds of foods, and it doesn’t have to be expensive. This module shows you how <http://eatsmart.org.au/>. Be inspired by our fun shopping video, and look in the Resources for a menu planner you can use to help plan your weekly shopping. Stella | **5. Eating out smart** | Hi *(Participant’s name)*, Stella here. *(Participant’s name)*, Don’t let diabetes stop you eating out or travelling! But can you keep up those healthy eating habits away from home? Module 5 will show you how, with useful ideas, e.g. choose vegetables or salads instead of chips to go with main meals (at least most of the time). Take healthy snacks with you, instead of buying expensive junk food. Find out more in our new module, Eating Out Smart! <http://eatsmart.org.au/> |
|  | Hi *(Participant’s name)*, a great healthy eating strategy is to spend most (more than half) of your budget on these “Eat Most” foods: vegetables, fruits, wholegrain breads and cereals. *(Participant’s name)*, think about how much you spend on these now. To help, try our Module 2 activity <http://eatsmart.org.au/>. If someone else does the grocery shopping at your home, maybe you could do this activity together. Stella |  | Hi *(Participant’s name)*, by now you’ve hopefully set a few healthy eating (or drinking) goals. How are you progressing with these? If you are meeting them, well done! Try to keep up the healthy changes you’ve made, and set some slightly higher targets next time, until you reach the 2 and 5 fruit and veggie goals. If you’re not, don’t give up. Think about what makes it hard, and how you can make it easier next week. Stella <http://eatsmart.org.au/> |
|  | Hi *(Participant’s name)*, are you going well with your healthy eating goal for this module? If so, great! If not, what are your challenges? How can you overcome them? If you haven’t already, try setting a goal now, e.g. add another serve of vegies or fruit towards that 5 and 2 target, or spend more of your budget on “Eat Most” foods. Plan how you will do this. Record your goal and how-to plan <http://eatsmart.org.au/> |  | Hi *(Participant’s name)*, eating out? Instead of pies, pasties or hotdogs, try a hamburger or souvlaki filled with fresh salad. Frozen yoghurt is delicious and has much less fat than ice cream. For more great swaps, check out module 5 at the website <http://eatsmart.org.au/>. Perhaps, *(Participant’s name)*, you can set a goal to make one healthy food swap this week! Give it a try. Stella |
|  | Hi *(Participant’s name)*, we should spend around a third of our food money on “Eat Moderately” foods: lean meats, chicken without skin, fish, eggs, reduced-fat milk, yoghurt, cheese, nuts and seeds. How much do you spend on these now? Module 2 shows you how to work this out, step by step: give it a try <http://eatsmart.org.au/>. Also, make sure you check the Myths about diabetes and food in every module. Stella |  | Hi *(Participant’s name)*, when you’re in a hurry, it can be tempting to grab takeaway or frozen convenience meals, but a stir-fry with tasty, colorful vegetables and noodles can be faster, cheaper and healthier! Check out Ash and Lena in their module 5 video for some delicious budget shopping and cooking options! <http://eatsmart.org.au/> |
|  | Hi *(Participant’s name)*, spend less than 10% of your food money on “Eat Least” foods: sugary soft drinks, potato chips, sweets, biscuits, beer, wine and chocolate. How much do you spend on these foods, which often raise your blood sugars? As best you can, replace these with Eat Most foods or only eat them in small amounts, now and then. For tips on how, see <http://eatsmart.org.au/>. Stella |  | Hi *(Participant’s name)*, need more eating-out ideas? Eat fewer deep-fried items: instead, baked, grilled, boiled or steamed items are delicious and healthier. Get in the habit of ordering salad or vegetables to eat with your main meal. When travelling, try taking water, maybe with a twist of fruit, rather than buying soft drinks to drink. This will help keep your blood sugars on target, save money and let you enjoy your time more! See module 5 for more ideas <http://eatsmart.org.au/>. Stella |
|  | Hi *(Participant’s name)*, it’s Stella here. Need new ideas for tasty recipes on a budget? Each EatSmart module provides four delicious, low-cost recipes, based around vegies or fruits and other Eat Most foods. Aim to try one this week! You’ll find them near the end of each module. Remember, eating in a healthy way helps to smooth blood sugar levels. <http://eatsmart.org.au/> |  | Hi *(Participant’s name)*, have you tried Chinese cabbage, or carambola?! Check out vegetables and fruits that you don’t normally eat or are unfamiliar with. Maybe try a food style that is different from your own, and explore new ways to enjoy fruits, vegetables and other ‘Eat Most’ foods. We have more Smart ideas for you here, *(Participant’s name)*! <http://eatsmart.org.au/> Stella |
| **3. Shopping smart** | Hi *(Participant’s name)*, we’ve just unlocked a new module for you: Shopping Smart to Eat Smart! Check out all the great tips and recipes, including some easy snack ideas. Find out why choosing fruit and vegetables in season is a good option, and how to store them. Also enjoy our second video - Ash & Lena give lots of helpful food shopping hints and a recipe too! Stella <http://eatsmart.org.au/> | **6. Bringing it all together** | Hi *(Participant’s name)*, well done. You’ve reached the final EatSmart module! Module 6 brings it all together, with some extra hints for how to maintain your healthy habits into the future. You might be surprised how much you have learnt! <http://eatsmart.org.au/> If you haven’t already, test what you now know by doing the quizzes near the end of the module. Stella |
|  | Hi *(Participant’s name)*, Stella here. Drinking water means you can save money by not buying other drinks, like sugary soft drinks, which raise your blood sugars. Think about setting a goal to swap a less healthy drink for water every day this week. You could also try out a Shopping Smart goal – have a look at our Module 3 suggestions <http://eatsmart.org.au/> |  | Hi *(Participant’s name)*. Now you know that healthy eating doesn’t need to be confusing. Try to stick to the three key habits we’ve built here: eating 2 serves of fruit and 5 serves of vegetables every day, and drinking plenty of water. You might want to go back to earlier EatSmart modules and recipes for more ideas and inspiration, and pass on your favorite healthy eating ideas or recipes to family or friends. Stella <http://eatsmart.org.au/> |
|  | Hi *(Participant’s name)*, fresh vegetables and fruits are cheapest and most nutritious when they’re in season. But did you know that frozen and tinned foods can be just as nutritious and convenient? Aim to always keep supplies of these easy-to-store vegies and fruit on hand, ready to use in your delicious meals. Why not try another of our tasty recipes soon! <http://eatsmart.org.au/> Stella |  | Hello *(Participant’s name)*. Make healthy eating easier by planning ahead, sticking to a shopping list, and spending most of your budget on Eat Most foods. See Modules 2 and 3 for reminders <http://eatsmart.org.au/>. Keep trying to use wholegrain or wholemeal options whenever you can, and eat small amounts of healthy snacks, such as crunchy low-salt popcorn, to help keep your blood sugars within target range. Stella |
|  | Hi *(Participant’s name)*, we know that when you’re hungry, it’s tempting to reach for less healthy convenient foods. Keep temptation away by not having these foods at home. Instead, plan ahead to buy snacks like fresh fruit, wholegrain crackers with hummus, yoghurt, unsalted nuts and air-popped popcorn at your next shop. Suggestion/idea: these snacks can help to keep your blood sugar stable. See more great ideas here: <https://www.heartfoundation.org.au/recipes/category/snacks> Stella |  | Hi *(Participant’s name)*. If preparation time is an issue, tinned or frozen vegetables are really fast, easy and nutritious options. Some of our recipes let you use frozen vegetables or fresh ones – why not try both! You might like to watch the videos in modules 4 and 5 again for some great cooking options <http://eatsmart.org.au/>. Also, have a look in the Resources section of this module to find other important lifestyle habits that can help keep you feeling better. Stella |
|  | Hi *(Participant’s name)*, here are useful Smart Shopping tips: pre-plan your meals and write down the ingredients you need to buy on a grocery list. Then only buy what’s on the list. Also, if you can, save by stocking up on frozen and tinned fruits and vegetables when they are on sale. Remember to compare the cost of different sized products per 100g. Our Meal Planner can be found in the Module 2 Resources <http://eatsmart.org.au/> Stella |  | Hi *(Participant’s name)*, Stella here. Now that you’ve almost completed our EatSmart program, think about how you can keep up positive changes. Keep eating lots of vegetables & two fruits daily, to add wonderful color, flavor and nutrients to your meals. Save money by eating less processed food and drinking water instead of sugary drinks. Enjoy easy healthy snacks. Perhaps you can involve your family or friends to spread the word and support one another! <http://eatsmart.org.au/> |
|  | Hi *(Participant’s name)*, people with diabetes can also enjoy dessert! Learn more shopping tips and how to make a delicious dessert recipe with Lena and Ash in their third video <http://eatsmart.org.au/>. How did you go with your water goal this week? Can you continue to drink water instead of sugary drinks? Remember, this saves you money to spend on other things, too! Stella |  | Hi *(Participant’s name)*. Congratulations!! You’ve reached the end of the EatSmart program! By now, you’ve learnt to Eat, Budget, Shop, Cook and even Eat Out in a Smart way! If you like, you can go back to have another look at the modules. We really hope you’ve enjoyed the program and can keep up your positive eating habits into the future. We’ll be in touch soon to find out how you went. Thank you for being an important part of it and Happy Healthy Eating! Stella & the EatSmart Team. <http://eatsmart.org.au/> |
