# Supplementary material for: EatSmart, a Web-Based and Mobile Healthy Eating Intervention for Disadvantaged People With Type 2 Diabetes: Protocol for a Pilot Mixed Methods Intervention Study
Source: JMIR Res Protoc. 2020 Nov 6;9(11):e19488. doi: 10.2196/19488 (PMC7679211; doi:10.2196/19488)
Supplement: Multimedia Appendix 5 [file resprot_v9i11e19488_app5.docx]

| **outcomes** | **Measures** | **Response options** | **baseline** | **3 month** | **9 month** | **Source** |
| --- | --- | --- | --- | --- | --- | --- |
|  |  |  |  |  |  |  |
| **consumption of vegetables** | How many serves of:   1. Non-starchy vegetables do you usually eat per day? | 0=I don't eat vegetables, 1=<1 serve/day, 2=1 serve/day, 3=2 serves/day, 4=3 serves/day, 5=4 serves/day, 6=5 serves/day, 7=6 or more serves/day | X | X | X | Adapted from McLennan, the 1995 national nutrition survey or Originally developed |
|  | 1. Starchy vegetables do you usually eat per day? | 0=I don't eat starchy vegetables, 1=<1 serve/day, 2=1 serve/day, 3=2 serves/day, 4=3 serves/day, 5=4 serves/day, 6=5 serves/day, 7=6 or more serves/day |  |  |  |  |
|  | 1. Legumes do you usually eat per day? | 0=I don't eat legumes, 1=<1 serve/day, 2=1 serve/day, 3=2 serves/day, 4=3 serves/day, 5=4 serves/day, 6=5 serves/day, 7=6 or more serves/day |  |  |  |  |
| **consumption of fruits** | About how many serves of fruits do you usually eat per day? | 0=I don't eat fruit, 1=<1 serve/day, 2=1 serve/day, 3=2 serves/day, 4=3 serves/day, 5=4 serves/day, 6=5 serves/day, 7=6 or more serves/day | X | X | X | Adapted from McLennan, the 1995 national nutrition survey |
| **consumption of wholemeal/wholegrain bread, low-fat milk, water, soft drinks and other discretionary foods such as pastries, cakes, and sugar-sweetened beverages** | In the past 3 months, about how often have you eaten the following?   1. Potato crisps or other salty packet snack foods 2. Chocolate or lollies 3. Cake, doughnuts or sweet biscuits 4. Pies, pasties or sausage rolls 5. Fast food (e.g. McDonalds, KFC) 6. Pizza (not home-made) 7. Hot chips | 0=never or <1/month, 1=1-3 times/month, 2=once/week, 3=2-4 times/week, 4=5-6 times/week, 5=once/day, 6=2-3 times/day, 7=4-5 times/day, 8=6 or more times/day | X | X | X | Adapted from Ball et al. [58] |
|  | What type of bread do you usually eat? | 0= I don’t eat bread, 1= High-fiber white bread, 2=White bread, 3=Wholemeal bread, 4=Rye bread, 5= Multigrain/wholegrain bread | X | X | X |  |
|  | What type of milk do you usually drink? | 0= Whole milk, 1= Reduced fat milk, 2=Skim milk, 3= Other type of milk (please specify), 4= I don’t drink milk |  |  |  |  |
|  | About how many much plain water (still water, not  sparkling or soda water) in total do you usually drink each day? | 0=I don't drink water, 1=<1 serve/day, 2=1 serve/day, 3=2 serves/day, 4=3 serves/day, 5=4-5 serves/day, 6=6-7 serves/day, 7=8-9 serves/day, 8=10 or more serves/day |  |  |  |  |
|  | About how many serves of FRUIT JUICE do you usually I don't drink fruit juices drink per day? | 1=I don't drink fruit juice 2=<1 serve/day, 3=1 serve/day, 4=2 serves/day, 5=3 serves/day, 6=4-5 serves/day, 7=6-7 serves/day, 8=8-9 serves/day, 9=10 or more serves/day |  |  |  |  |
|  | About how much full-calorie SOFT DRINK do you usually drink each day? (DO NOT COUNT diet soft drink.) | 1=I don't drink full-calorie soft drink 2=<1 serve/day, 3=1 serve/day, 4=2 serves/day, 5=3 serves/day, 6=4-5 serves/day, 7=6-7 serves/day, 8=8-9 serves/day, 9=10 or more serves/day |  |  |  |  |
|  | About how much DIET SOFT DRINK (i.e. no-calorie or sugar-free fizzy soft drink) do you usually drink each day? | 1=I don't drink diet soft drink 2=<1 serve/day, 3=1 serve/day, 4=2 serves/day, 5=3 serves/day, 6=4-5 serves/day, 7=6-7 serves/day, 8=8-9 serves/day, 9=10 or more serves/day |  |  |  |  |
| **Dietary self-efficacy** | How much do you agree or disagree with the following?   1. I feel confident that I can plan meals or snacks with more fruit during the next week 2. I feel confident that I can buy more vegetables the next time I shop 3. I feel confident that I can buy more fruit the next time I shop 4. I feel confident that I can plan meals with more vegetables during the next week 5. I feel confident that I can eat fruit or vegetables as snacks 6. I feel confident that I can add extra vegetables to dishes such as casseroles, stews, pastas and stir-fries 7. I feel confident that I can eat two or more servings of vegetables at dinner 8. I feel confident that I could replace less healthy foods like cakes, pastries and chocolates with healthier options 9. I feel confident that I could replace soft drink with water | 0=agree, 1=neither agree nor disagree, 2=disagree | X | X | X | Adapted from Townsend et al. [59] |
| **Dietary self-efficacy (Part 2)** | 1. How do you feel about the following questions? 2. How confident do you feel about being able to cook from basic ingredients including vegetables? 3. How confident do you feel about following a simple recipe that includes fruits and/or vegetables? 4. How confident do you feel about tasting fruits and vegetables that you have not eaten before? 5. How confident do you feel about preparing and cooking fruits and vegetables that you have not cooked with before? | 1= extremely confident, 4=unsure, 7= not at all confident | X | X | X | Adapted from Wrieden et al. [60] |
| **Barriers to healthy eating** | How much do you agree or disagree with the following statements?   1. I feel that fruit is too expensive 2. I feel that fruit is not always available 3. I feel that fruit is not tasty 4. I feel that vegetables are not always available 5. I feel that vegetables are not tasty 6. I feel that the fresh fruit and vegetables in my neighborhood are of a high quality 7. I feel that if I buy more vegetables, they might be wasted 8. I feel that if I buy more fruit, it might be wasted | 0=agree, 1=neither agree nor disagree, 2=disagree | X | X | X | Adapted from Townsend et al. [49] |
| **Barriers to healthy eating (Part 2)** | How much do you agree or disagree with the following statements?   1. I feel that I have enough knowledge about how to prepare/cook vegetables 2. I like the taste of full-calorie (regular/non-diet) soft drinks too much to cut down on drinking these 3. I like to drink water 4. I feel that vegetables are time-consuming to prepare 5. Members of my household like the taste of full-calorie (regular/non-diet) soft drink too much to cut down on buying/drinking these 6. Members of my household like to drink water 7. I feel that fruit is not liked by members of my household 8. I feel that vegetables are not liked by members of my household | 0=agree, 1=neither agree nor disagree, 2=disagree | X | X | X | Adapted from Ball et al. [11] and Townsend et al. [59] |
| **Program feasibility** | Numbers recruited and retained  Objective indicators of engagement with the program  Numbers using the website; log-in frequency and duration; pages visited; SMS messages read/engaged with |  | X | X | X | Adapted from Fritz et al. [61] and modified to meet the specify needs of this study |
| **Program Appeal, usability and participants satisfaction** | Over the past 12 weeks, did you look at the Eat Smart website? | 0= Yes, 1= No, 3= Don’t know |  | X | X | Original |
|  | How useful did you find the website? | 1= Not at all useful, 5= Extremely useful |  |  |  |  |
|  | How many of the six Eat Smart modules did you look at? | 0= All, 1= About three-quarters, 2= About half, 3= About a quarter, 4= None |  |  |  |  |
|  | What were the MOST useful parts of the website? | Descriptive text |  |  |  |  |
|  | What were the LEAST useful parts of the website? | Descriptive text |  |  |  |  |
|  | What was one main message you remember from looking at the website? | Descriptive text |  |  |  |  |
|  | In the past 12 weeks, did you receive phone (SMS text) messages from us on your mobile phone? | 0= Yes, 1= No, 3= Don’t know |  |  |  |  |
|  | If YES, how useful did you find the phone messages? | 1= Not at all useful, 5= Extremely useful |  |  |  |  |
|  | How many of the phone messages did you read? | 0= All, 1= About three-quarters, 2= About half, 3= About a quarter, 4= None |  |  |  |  |
|  | If you didn't read them all, can you please let us know why? | Descriptive text |  |  |  |  |
|  | What were the MOST useful parts of the phone messages? | Descriptive text |  |  |  |  |
|  | What were the LEAST useful parts of the phone messages? | Descriptive text |  |  |  |  |
|  | What was one main message from the phone messages that you remember? | Descriptive text |  |  |  |  |
|  | Have you changed the way you buy, cook or eat food after taking part in the Eat Smart study? If YES, can you please explain how you have changed the way you buy, cook or eat food? | 0= Yes, 1= No, 3= Don’t know  Descriptive text |  |  |  |  |
|  | Overall, what did you like most about being involved in the Eat Smart study and why? | Descriptive text |  |  |  |  |
|  | Do you have any suggestions for how we could improve this study? | Descriptive text |  |  |  |  |
|  | Interview with HCPs | Open ended questions |  | X |  |  |
|  | Interview with patients | Open ended questions |  |  | X |  |
| **Demographic data** | Country of birth | Australia, India, Vietnam, Malta, Italy, others | X |  |  |  |
|  | The highest education level | 0= Year 10 or less, 1= Year 12, 2= Trade/Certificate/Apprentice, 3= University degree, 4=Higher degree |  |  |  |  |
|  | Gender | 0= Female, 1= Male, 2= Identify as other |  |  |  |  |
|  | Age | Date of birth |  |  |  |  |
|  | Marital status | 0= Single, 1= De facto, 2= Married, 3= Divorced |  |  |  |  |
